# A shadow in the treatment of acute leukemia: lineage switch

**DOI:** 10.1097/BS9.0000000000000220

**Published:** 2025-03-20

**Authors:** Qiaoyi Zhou, Ying Wang

**Affiliations:** a State Key Laboratory of Experimental Hematology, National Clinical Research Center for Blood Diseases, Haihe Laboratory of Cell Ecosystem, Institute of Hematology & Blood Diseases Hospital, Chinese Academy of Medical Sciences & Peking Union Medical College, Tianjin 300020, China; b Tianjin Institutes of Health Science, Tianjin 301600, China

**Keywords:** Acute leukemia, Lineage switch

## Abstract

Lineage switch is a rare phenomenon in which acute myeloid leukemia (AML) transforms into acute lymphoblastic leukemia (ALL) and vice versa, sharing the same clonal origin. It is more common for AML to relapse as ALL. Cytogenetics, microenvironment, and preceding therapies are associated with lineage switch. Since the etiology of lineage switch is unclear, presumptions include clonal selection, pluripotent stem cells, and differentiated cell trans-differentiation or re-differentiation. The key point for diagnosing lineage switch is that the relapsed tumor originates from the common cell of the primary leukemia, although it is occasionally derived via clonal evolution. It is very important to distinguish lineage switch from other illnesses, such as secondary leukemia or the blast phase of chronic leukemia. Although direct treatment of the present lineage results in an improved prognosis, the outcome of these patients remains poor, with low survival and rapid progression. Hematopoietic stem cell transplantation can extend survival. Lineage switch risk-adapted management stratification may be beneficial for detecting relapse and more promptly provide suitable therapy. Efficient and toxicity-restricted therapy is being developed to improve the very poor prognosis.

## 1. INTRODUCTION

Lineage switch is a rare phenomenon that occurs in acute leukemia, in which one lineage sharing the same clonal origin converts into another lineage during the course of the disease. It is more common for acute lymphoblastic leukemia (ALL) to relapse into acute myeloid leukemia (AML), particularly the transformation between B-cell ALL and monoblastic leukemia. A multicenter prospective study by Zhou et al^1^ reported that among 33 patients with lineage switch, 28 were diagnosed with ALL and relapsed to AML, and 25 were B-cell ALL. In addition, regardless of primary or second leukemia presenting as a myeloid lineage, most were monocytic or myelomonocytic differentiation (21/33, 64%),^1^ although infrequently, there have been several reports of AML switching to ALL.^2–18^

Most patients undergoing lineage switch are children, particularly those with mixed lineage switch leukemia (*MLL*) gene rearrangement. The incidence of lineage switch is approximately 0.6% among children with acute leukemia.^8^ A recent study following up patients with lineage switch showed a median age of 8.4 years (range 1 day to 76.5 years) at primary diagnosis, and a median age of 11 years for developing lineage switch.^19^ However, the incidence may have been underestimated due to inaccessibility and lower precision of testing techniques, such as flow cytometry (FCM) or polymerase chain reaction (PCR). Currently, with the widespread use of antigen detection or cellular molecular techniques and the burgeoning of immunotherapy, reports of patients with lineage switch have increased.^20–41^

The duration between diagnosis and lineage switch is usually relatively short, with a median of 2.2 years,^17^ except for several years reported in a few patients.^23,42^ This shows that primary leukemia in children is more likely to be T-cell ALL or AML than in adults, and the interval between diagnosis and lineage switch is longer.^1^

Lineage switch can occur in remission or during treatment^8^ and 79% of lineage switch occurs after complete remission.^1^ Switched leukemia can be discovered in the bone marrow, central nervous system, or outside the spinal cord.^19^ The incidence of lineage switch arising in the bone marrow is 6.7%.^17^

The prognosis of lineage switch is very poor,^23^ highlighting the necessity for a deeper understanding of this phenomenon. According to previous studies, treatment of the current lineage resulted in better outcomes.^17^ Given the susceptibility of acute leukemias to lineage switch, a molecular test for recognizing tumor cells may still yield negative results during relapse.^43^ Thus, more methods to estimate disease burden are required. Meanwhile, for patients with leukemic cells that have higher lineage plasticity, monitoring of multiple cell lineage markers is recommended to more promptly identify lineage switch.

## 2. RISK FACTORS

### 2.1. Cytogenetics

*MLL* rearrangement (*MLL*r) is the most common chromosomal alteration detected in lineage switch.^1^ *MLL*r-related leukemia, a major cause of infant ALL, accounts for 5% to 10% of acute leukemias. *MLL* is above 92 kb long and contains at least 37 exons located on chromosome 11q23. MLL protein, the translating product of *MLL*, is a large 430 kDa protein containing histone methylase domains. *MLL* maintains *HOX* expression, activates *HOX-A* downstream expression, and stimulates immature stromal cellular proliferation during development and differentiation of hematopoietic stem cells (HSCs).^44,45^

Compared with primary leukemia cells, secondary leukemia cells have a more complex karyotype, suggesting genetic evolution in lineage conversion. As plasticity increases when chromosome 11 mutates and the MLL fusion protein is present, a lineage switch or mixed lineage leukemia is more likely to be present in patients harboring *MLL*r. *MLL*r predominantly results from a translocation between chromosomes 4 and 11, followed by t(9;11) and t(11;9). Other rare aberrations include t(2;11), t(10;11), and inv(11).^1^ Several precursors of MLL fusion protein have been reported, which can be categorized into 4 types: MLL-AEP, including MLL fused with the AF4/ENL/P-TEFb family; MLL-AF10 family; similarity with the MLL active form; and MLL dimer domain. MLL fusion protein may induce leukemogenesis by reactivating an early gene program rich in CpGs and constitutively stimulating HSC-programmed gene expression.^46^ Studies have reported that transducing MLL fusion protein into immature hematopoietic progenitor cells via a retrovirus can constitutively express the HSC programming gene. These cells cultured with myeloid cytokines continue to proliferate and differentiate, eventually leading to leukemia.^47^ A comparison of the genetic landscape of 2 groups of patients with lineage switch categorized based on the presence or absence of *KMT2A* mutation showed that the latter group had more features of chronic myeloid malignancy, suggesting a pivotal role of KMT2A in breaking the barrier of cell lineage.^1^

Patients with acute leukemia harboring *MLL*r may accumulate secondary chromosomal abnormalities during treatment, resulting in new mutations and the induction of lineage switch. Studies have shown that the karyotype detected when there is a lineage switch is always more complex, and the tumor more often carries at least 3 chromosome abnormalities.^1,48^ Nakajima et al^4^ reported a case in 2022 in which a child was diagnosed with AML and *KMT2A-MLLT3* rearrangement. During therapy, *PAX5* mutates, which often occurs in children with B-cell precursor ALL (BCP-ALL), and the disease relapses as BCP-ALL. Relapsed cancer is characterized by a profile closer to that of BCP-ALL with *PAX5* mutation rather than *KMT2A* rearrangement.^4^ This suggests that cells carrying *MLL*rs may be more genetically unstable; thus, they are more likely to mutate and cause lineage switch.

However, there are some reports on *MLL*r leukemia that develop lineage switch without clonal evolution, suggesting that factors other than genetic change can target lineage switch.^8,25,49^ Among them, epigenetics may play a role, given that the accessibility of genome chromatin and transcription factor binding sites in tumor cells change during lineage switch.^50^ MLL fusion protein is different between AML and ALL,^45,51^ as KMT2A-AFF1 translocation is almost only present at diagnosis as B-cell ALL, and the KMT2A fusion form plays an important role in determining the binding site of the oncogene. The KMT2A oncoprotein binds to different domains in AML and ALL. It has been shown that during lineage switch, KMT2A-AFF1 activates a series of target genes that are different from other *KMT2Ar* AML target genes at primary diagnosis.^52^ The target genes include many, such as *SRG*N and *LYS*T that are involved in the production and secretion of hemopoietic granules and are normally expressed in granulocytes, particularly in neutrophils. The expression of oncogenes and their binding sites is dynamic. The change in the KMT2A proto-oncoprotein from a structural role to a non-structural role in the cell promotes the development of lineage conversion. It is suggested that the intracellular *KMT2A* proto-oncogene in patients with *MLL*rs promotes the differentiation of leukemia cells to one lineage, while the leukemia cells tend to differentiate to another lineage; lineage transformation occurs when the treatment affects the expression level of the *KMT2A* proto-oncogene.^53^ Some patients with lineage conversion have multiple lineage switches during the course of the disease, which may also be related to the fluctuating expression levels of proto-oncogenes.^5,8,36,49,54,55^

In addition, *CEBPA*, *CBFB-MYH44* type A fusion, *PAX5*, *BCOR, BCORL1*, t (9; 22)/*BCR-ABL*, -9p-/*CDKN2A* deletion, *RUNX1*, *EZH2* and 5q- in a complex karyotype, -7/7q-, complex duplication, +13,17p11.2 mutation, partial chromosome 6 deletion, t(12; 19)/*TCF::ZNF384*, t(5; 14)/*TCLX3::BCL11b* may also be related to lineage switch.^1,4,7,25,30,38,39,43,55–57^ Table 1 shows some reported cytogenetic mutation in lineage switch. A lineage switch has been reported, in which a patient with B-cell ALL and a normal karyotype at diagnosis gained mutations during therapy, including *NRAS(G12D*) hot spot mutation, *CDKN2A*, *PAX5,* and *ZCCHC7* aberration. Lineage switch occurs after a small part of the chromosome involving loss of remaining *PAX5* and *ZCCHC7* alleles, causes a biallelic *PAX5* and *ZCCHC7* alteration.^21^ As *PAX5* is a master transcription factor of B-cell commitment, its mutation may retain cells to further develop as B-lineage and result in myeloid differentiation.

**Table 1:** Detailed information of some reported lineage switch leukemias (- refers to unavailable).

Reference	Cytogenetic aberrant		First diagnosis	Second diagnosis
	At diagnosis	At lineage switch		
55	Complex karyotype (44,X,-X,add(1)(p13),add(2)(q21),-4,-5,-10,del(11)(q)?,-12,-14,-17,-18,+r1,+mar1,+mar2,+mar3,+mar4,+mar5[3]/46,XX[4])	Complex karyotype (45,X,-X,add(1)(p13),add(2)(q21),-4,-5,-8,-10,add(11)(q13),-12,-13,add(17)(p11.2), +mar1,+mar2,+mar3,+mar4,+mar5,+mar6[1]/43,idem,-7,-add(17),+add(17)(p11.2),-mar6)	B-ALL	AML
25	*-*	*KMT2A::AFF1*,+6,der(15)add(p13)	BCP-ALL	AML
25	Normal	*TCF::ZNF384*	BCP-ALL	MPAL (B + myeloid)
25	*KMT2A::MLLT1*,+6	-	BCP-ALL	MPAL (B + unclassifiable)
25	*KMT2A::AFF1*,der(13)c	*KMT2A::AFF1*,der(13)c,+der(4)t(4;11)(q21.3-q22.1;q23.3)	BCP-ALL	MPAL (B + myeloid)
25	*KMT2A::MLLT1*	*KMT2A::MLLT1*	BCP-ALL	AML
25	*KMT2A::AFF1,*+22	*KMT2A::AFF1,*complex karyotype(56,XX,+X,+der(4)t(4;11)(q21.3-q22.1;q23.3,+der(4)t(4;11)(q21.3-q22.1;q23.3),+der(6),+der(6),+7,+8,der(11)t(4;11) (q21.3-q22.1;q23.3),+13,+17,+22[14]/46,XX[1])	BCP-ALL	AML
58	*KMT2A*-*AFDN*;Complex karyotype(47,XX,t(6;11)(q27;q23),+der(12)t(12;18)(p13;q12),der(18)t(12;18),+der(20)(p?;-21)	*KMT2A*-*AFDN*	ETP-ALL	AML
59	Normal	*TP53*	B-ALL	AML
20	*KMT2A::AFF1*	*KMT2A::AFF1*	B-ALL	MPAL (B + myeloid)
21	*NRAS,CDKN2A,PAX5,ZCCHC7,KMT2D*	*NRAS,CDKN2A,PAX5,ZCCHC7, KMT2D*	B-ALL	AML
24	normal	*TP53,RUNX1,PHF6*	T-ALL	AML
57	*PAX5,NRAS,CDKN2A*,+6,+7	*PAX5,NRAS,CDKN2A*,+6,+7	B-ALL	AML
5	*KMT2A-PTD*	*KMT2A-PTD*	AML	MPAL (B + myeloid)
27	*KMT2A::AFF1*	*KMT2A::AFF1,TP53,RUNX1*	B-ALL	AML

ALL = acute lymphoblastic leukemia, AML = acute myeloid leukemia, BCP = B-cell precursor, MPAL = mixed-phenotype acute leukemia.

However, there are also cases of lineage transformation with a normal karyotype and gene mutation screening at initial diagnosis and recurrence,^10,15–17,28^ suggesting that there are other factors causing lineage switch in addition to cytogenetic alterations.

### 2.2. Tumor microenvironment

The tumor microenvironment can affect the direction of cell differentiation and efficacy of therapeutic drugs, thus participating in lineage switch. Studies have shown that MLL-AF4 fusion products can proliferate as pro-B-cell ALL but induce AML when pretreated with myeloid-related cytokines with retained lymphoid-lineage potential.^60^ Culturing a human leukemic cell line with t(4,11), cells, which are characterized as myeloid/pre-B-cell bi-phenotype, differentiate along the myeloid lineage with a low concentration of an interleukin 6 (IL-6) supplement, indicating that cytokines can affect the direction of differentiation.^61^ In vitro experiments and mouse models culturing PAX 5-deficient pro-B cells with different cytokines can direct commitment in different directions. If IL-7 is added, the cells will continue to differentiate along the B lymphoid lineage, while the addition of macrophage-colony stimulating factor can direct commitment of macrophages.^62^ Besides, RNA analysis of a leukemia switch from T-cell ALL to AML shows amplification of the IL-7 receptor, suggesting that by upregulating specific receptors, cells may be more sensitive to some cytokines, thus influencing the direction of differentiation.^24^ The tumor microenvironment can also affect the tumor-killing effect of chemotherapy drugs, affecting clonal selection or gene expression in tumor cells.

### 2.3. Preceding therapy

A prospective cohort of childhood acute leukemia had a median of 7.8 (range 6–10) drugs received before lineage switch,^17^ thus making it difficult to assess the impact of frontline treatment-specific medication on the development of lineage switch. The earliest reported lineage switch occurred in patients receiving chemotherapy.^18,63^ The associated chemotherapeutic drugs, such as topoisomerase inhibitors, predispose to genetic instability^64^ and cause more genetic changes or affect the expression of differentiation-related genes, eventually inducing lineage switch. Lee et al^11^ reported a patient with acute leukemia that was primarily diagnosed as AML M4 that switched to bi-phenotypic acute leukemia after chemotherapy using a topoisomerase II inhibitor. The gradual acquisition of cytogenetic alterations and lineage conversion explains the occurrence of lineage transformation, which may be related to secondary leukemic cells and accumulation of genetic abnormalities. In addition, chemotherapy drugs such as adenosine deaminase inhibitors are also associated with a phenotypic switch.^17,63,65^

With the development of immunotherapy, reports of lineage switch after monoclonal antibody or chimer antigen receptor T-cell (CAR-T) therapy have been notably increasing. Clonal selection, epigenetic adaptation under selection pressure, or changes in the tumor microenvironment may contribute to these phenomena, with extreme selection pressure imposed by immunotherapy compared with conventional therapies. Moreover, common adverse effects of immunotherapy include the cytokine release syndrome, in which the IL-6 level increases significantly. In vitro experiments showed that IL-6 promoted the differentiation of B-cell ALL cell lines carrying *KMT2Ar* into the myeloid lineage, which may be related to phenotypic conversion.^61^ The incidence of lineage switch in children and young refractory/relapsed (R/R) patients with B-cell ALL can reach approximately 3%, accounting for 7.4% of relapse after infusion CAR-T therapy against cluster of differentiation 19 (CD19).^23^ Similarly, during blinatumomab therapy for pediatric patients with BCP-ALL, 3.3% of the children had lineage switch.^25^ Another study reported that among 25 patients primarily presenting with B-cell ALL, 15 received immunotherapy before lineage switch.^1^ The study showed a median of 1.6 months (range, 7 days to 35.5 months) from the most recent immunotherapy to the onset of lineage shift.^19^ The prevalence of lineage switch has also been observed in real-world usage.^26^ Table 2 summarizes the relevant risk factors to lineage switch.

**Table 2:** Risk factors of developing lineage switch.

Risk factors	Examples
Cytogenetics	Genetic aberrant (eg, *MLL-r, BCR-ABL*)
	Epigenetic changes
Tumor microenvironment	Cytokine influence
	Effect on drug resistance
Preceding therapy	Chemotherapy
	Immunotherapy

## 3. ETIOLOGY PRESUMPTION

### 3.1. Clone selection

It is hypothesized that patients with acute leukemia and a lineage switch have the same leukemia lineage of the same clonal origin; however, existing detection techniques are unable to identify this smaller population of cells. Although treatment eradicates the largest population of cells, those committed to other lineages are insensitive to the regimen and expand, contributing to lineage transformation.^66^ Clinically, several patients with lineage conversion have 2 lineages of cells during therapy or even at initial diagnosis. The numbers of cells in the 2 groups fluctuated during the disease process along with the instant development of phenotypic conversion, explaining clonal selection.^5,8,17,25,49,56^ In addition, analysis of tumor cells from patients with lineage switch showed that many of the subclone mutations present at initial diagnosis gradually decreased at relapse. One patient with ALL relapsed in the bone marrow after remission, with relapsed leukemia cells resistant to salvage ALL treatment; some (23%) appeared weakly positive for myeloperoxidase (MPO) staining. After applying adenosine deaminase inhibitor 1 week after lineage switch, 60% of the bone marrow blast cells were strongly MPO positive, according to the transformation and amplitude of leukemia cells insensitive to the original treatment.^17^ A study of ALL antigen receptor gene rearrangement PCR in 27 patients showed 13 relapses as an obvious new clonal subtype (not detected by conventional testing). By tracking the specific molecular markers of the clonal subtype, 8 relapse samples of 12 informative patients supported to be identical with the initial diagnosis. Comparing the relationship between the subtype and treatment reveals that the recurrent subtype is insensitive to treatment, supporting the hypothesis that clonal selection plays an important role in the recurrence of acute leukemia.^67^ Single-cell multiomics analysis of paired leukemia samples with a primary diagnosis of BCP-ALL, which has a later phenotypic switch, shows that the presence of numerous myeloid cells at initial diagnosis and the respective activation of lineage-specific transcription factors in myeloid or B lymphoid primed blast cells, suggesting that lineage switch may be the result of clonal selection.^68^

However, a child with t(4; 11) (q21; q23) was first diagnosed with AML M5 as a congenital leukemia and still converted to ALL 3 weeks after diagnosis without cytotoxic agent adopted,^14^ indicating that lineage transformation may be the result of the natural progress of the disease rather than selection.

### 3.2. Multipotential leukemia stem cells

The same clonal origin between the first and second leukemia in lineage switch contributes to the possibility of common leukemic stem cells. In some patients with lineage switch, leukemia cells simultaneously express 2 surface lineage molecules at primary diagnosis.^5,9,13,14,29,30,56,58,69,70^ For instance, Krawczuk-Rybak et al^14^ and Boeckx et al^13^ reported lineage conversion where AML was considered to be the first leukemia with bone marrow monoblastic cells co-expressing CD19^9^ indicating the possible existence of leukemic stem cells with myeloid/lymphoid differentiation bi-potential, which is bi-phenotypic initially, gradually losing one lineage surface molecular marker during differentiation and transforming into other lineages. In addition, a study for AL patients with t(4;11) reported a case in which MPO activity elevated in lymphoid-appearing blasts and some neutrophil myelocytes with Auer rods were detected, implying the existence of multipotential leukemia stem cells.^71^ Single-cell multiomics analysis of fetal BCP-ALL samples revealed that a small group of lymphomyeloid-primed progenitor (LMPP)-like naive cells were present before and after lineage switch, and the LMPP-like naive cells were significantly associated with myeloid blast cells, suggesting that patients with lineage conversion may have precursor cells that can differentiate into the myeloid or lymphoid lineage, and lineage transformation occurs when the transcription factor expression level is reprogrammed.^68^ An analysis of the fluctuation in the cellular signature between primary leukemia and lineage switch had the same outcome.^50^ Precursor cell’s commitment to myeloid/lymphoid multilineage has been successfully isolated in fetal liver in vitro.^72^ Bone marrow-derived pro-/pre-B-cells showing partial loss of PAX5 function have bi-phenotypic-primed and upregulated myeloid lineage genes, in vivo and in vitro.^73,74^

### 3.3. Trans-differentiation and re-differentiation of mature cells

**Figure 1.**
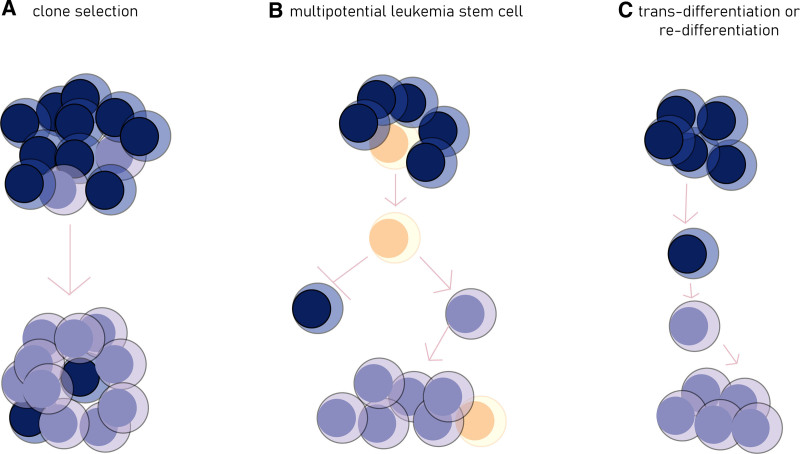
Clone selection, the existence of multipotential leukemia cells, and trans-differentiation or re-differentiation of mature cells are the hypothesized mechanism lineage switch. The blue cells represent primary leukemia cells. The purple cells represent secondary leukemia cells. The yellow cells represent multipotential leukemia stem cells.

With continuous progress, the theory of irreversibly differentiated cells is gradually being questioned. Epithelial-mesenchymal cell transformation is occasionally discovered in inflammation and malignant conditions, confirming that differentiated cells can switch to another type of differentiated cell through de-differentiation, re-differentiation, or direct trans-differentiation.^75^ Examination of *BCR* rearrangement in tumor cells from patients diagnosed with ALL and relapsing as AML revealed that some of the relapsed myeloid cells express *BCR* rearrangement, probably trans-differentiating or re-differentiating from the ALL clone at the initial diagnosis.^21^ The accumulating clonal frequency of immunoglobulin sequences, along with the myeloid leukemia relapsed from B cell-ALL, was also detected.^30^ Moreover, the above mechanisms were found in samples from patients who relapsed after treatment with blinatumomab or CD19 CAR-T cell, suggesting that direct trans-differentiation may be one of the mechanisms causing immune escape.^30,50^ The conversion of differentiation may be associated with genetic reprogramming. During the lineage switch from ALL to AML, upregulation of myeloid-related genes and lower expression of lymphoid lineage-related genes have been detected.^30,50^ Figure 1 illustrates the possible mechanism developing lineage switch.

In addition, risk factors induce the development of lineage switch through one or more presumptive pathways of transforming leukemia lineages.^76^

## 4. DIAGNOSIS

Patients are always admitted to hospital for symptoms such as fever, fatigue, respiratory tract infections, or hemorrhage.^5,16,55,58,59^ Physical examination may reveal paleness; liver, spleen, or lymph node enlargement; visible sources of skin hemorrhage; or nodules.^5,48,57,65,77^ Occasionally, central nervous system involvement is observed.^65^ Abnormalities in at least 1 lineage and an increased lactate dehydrogenase level are usually present in the peripheral blood.^48,55,65,77^

The key point in diagnosing lineage switch is that primary and secondary leukemia cells share the same clonal origin, which can be confirmed by identical chromosome/gene variation or immunoglobulin/T-cell receptor rearrangement. Therefore, it is recommended that such information be obtained from patients with primary and suspected relapse using fluorescent in situ hybridization, next-generation sequencing, or other techniques.

The treatment must differ from that used for secondary acute leukemia and the blast stages of chronic blood diseases. Secondary treatment-related acute leukemia is usually observed in older patients, and chromosome/gene variants or immunoglobulin/T-cell receptor rearrangements are often very different between initial and recurrent samples. Treatment-related acute leukemia is often AML and less commonly harbors Auer bodies. Leukemia previously treated with alkylating agents usually has a longer interval,^32^ approximately 5 to 7 years, between exposure and occurrence, and always presents with deletion of the long arm of chromosome 5 or 7. Moreover, DNA topoisomerase II inhibitor-related secondary acute leukemia usually has a shorter duration of approximately 2 years and is associated with *MLL* gene rearrangement.^78^ The incidence of secondary acute leukemia is higher compared with lineage switch. A prospective study in children with acute leukemia showed that the incidence of lineage conversion was 0.6% and 1.4% for secondary leukemia.^8^

Transformation to myeloid or lymphoid lineage is often observed during the blast phase of chronic myeloid leukemia (CML). Patients with CML are primarily diagnosed with AML M5, which later converts to ALL. However, CML usually carries the Philadelphia chromosome or presents as *BCR/ABL* positive. Hepatosplenomegaly and eosinophilia are often observed.

## 5. TREATMENT AND PROGNOSIS

Due to the occurrence of lineage switch, the markers used to monitor loading illness may remain negative when relapse occurs, so measurable residual disease (MRD) monitoring strategies should be optimized for earlier detection of relapse. If there are gene mutations, the tumor burden can be assessed by detecting the mutated gene using PCR-MRD instead of only FCM, given that the tumor can be FCM-MRD-negative but PCR-MRD can still be positive in lineage switch.^43,56^ At the same time, for patients at high risk of lineage switch, such as those with specific gene mutations, the expression level of multiple lineage molecular markers can be regularly tested. As patients with ALL carrying *AFF1-ALL* ectopic rarely express CD10, monitoring of the *MLL* gene when facing CD10-negative ALL is recommended.^50^ The study reported that 65.4% of children with CD371 (+) BCP-ALL had transient transformation to monocytic leukemia during induction treatment, and the expression of this molecule in leukemia was not downregulated during treatment.^79^ This suggests that CD371 may be used as an indicator to assess the risk of lineage switch at initial diagnosis and subsequent recurrence. The use of BCR/TCR clonality analysis in MRD monitoring is unreliable because several or even complete clonal markers are stable during lineage switch but may be substituted as the disease progresses.

Patients with lineage switch have a poor prognosis and aggressively progressive disease, with the vast majority of deaths resulting from the progression of the disease or complications of treatment, and only a few patients survive. A recent large prospective study of patients who developed lineage switch showed that only 17% survived in remission, with 87.5% receiving consolidation hematopoietic stem cell therapy (HSCT).^19^ The median survival time from initial diagnosis was approximately 12.3 months, and 2.3 months from the onset of lineage switch.^1^

Choosing a regimen based on the current spectrum provides a better treatment response while combining ALL and AML treatments simultaneously does not appear to reduce the incidence of lineage switch.^17^ However, for patients with specific mutations, the original lineage treatment regimen may be more effective. A study of 726 pediatric patients with BCP-ALL showed that 5 patients with the P80R mutation subtype of the *PAX5* gene developed lineage switch.^43^ This subtype is more responsive to prednisone and has a good prognosis. Since most patients with acute leukemia carrying this mutation will eventually develop B-cell ALL, a lymphoid-based regimen is recommended.^56^ Mallik et al^57^ reported an older female patient with *PAX5 p.P80R*, *NRAS p.G12S* alteration, *CDKN2A/B* biallelic deletion, *IKZF1* duplication, and an initials diagnosis of B-cell ALL. At the time of hormone and blinatumomab therapy, the lineage transformed on the eighth day after the initial diagnosis, which converted to AML M5. After 7 + 3 induction chemotherapy, she developed secondary hemophagocytic lymphohistiocytosis, infection, and other complications, and died due to adverse reactions to chemotherapy. The *PAX5 p.P80R*, *NRAS p.G12S* mutation, *CDKN2A/B* biallelic deletion, and *IKZF1* repeat remained at the time of lineage conversion, but the number of *PAX5 p.P80R* mutations significantly decreased, suggesting that the mutation is sensitive to hormonal therapy.^57^ However, genes such as *NRAS*, *TP53*, and *WT* do not appear to be involved in lineage switch.^1^

HSCT in patients undergoing lineage transformation can prolong overall survival. A study showed that in 8 of 33 patients with lineage switch who received HSCT after secondary leukemia, 7 with available data achieved complete remission, while only 1 of the remaining 19 patients who did not receive HSCT achieved complete remission (*P* < 0.001), suggesting that HSCT after lineage conversion can improve the outcome of these patients.^1^ A retrospective study using CD19 CAR-T therapy for patients with R/R B-cell ALL showed that none of the patients with KMT2Ar who received consolidation HSCT developed lineage switch,^23^ suggesting that HSCT consolidation therapy could be prioritized for patients at high risk of lineage switch with *MLL*r.

Owing to the fact that CD33 is expressed significantly higher in patients with AML, chromosome 11q23 translocations, and gemtuzumab ozogamicin (GO) treatment targeting CD33, may acquire clinical benefits. A randomized Phase III trial revealed that induction of GO can improve the outcomes of patients with *MLL*r and can even maintain a superior effect in the post-HSCT setting.^80^ Targeted drugs for *MLL*r involving signal pathway, such as revumenib, may be used to treat patients with *MLL*r. However, some studies have shown that epigenetic resistance mechanism to MLL fusion protein-targeting drugs is identical to that possibly causing phenotypic conversion,^53^ suggesting that these targeted drugs may have adverse effects in patients with lineage switch and may even accelerate the process. In addition, preclinical studies using FLT3-targeted CAR-T therapy for KMT2Ar ALL have shown benefits because *KMT2A* rearrangement highly expresses FLT3. The study also used cells acquired from a patient with lineage switch to create paired AML and ALL patient-derived xenograft models and the model was treated with FLT3 CAR-T. The results show that CAR-T therapy has equivalent inhibition of leukemia cell proliferation, even where few FLT3 receptors are expressed on the surface of transformed AML leukemia cells.^81^ Therefore, for patients with ALL and *KMT2Ar*, the therapeutic range of FLT3 CAR-T therapy can cover the initial tumor cells and the leukemia cells with lineage conversion, which may significantly improve the clinical outcomes of these patients.

## 6. CONCLUSION

Lineage switch, characterized by complete cell fate conversion from one lineage to another, is a rare phenomenon in the course of acute leukemia that often occurs in pediatric patients. In recent years, with the widespread application of molecular surface antigen detection technology and the emergence of immunotherapy, the number of patients identified with lineage switch has increased significantly, highlighting the pressing requirement to fully comprehend the mechanisms underlying this process.

Patients with lineage switch have a very poor prognosis, therefore, it is important to discriminate at-risk patients to stratify their management, improve disease burden monitoring methods to promptly identify relapse and lineage switch, and explore toxicity-limited and effective prevention and treatment strategies to improve their outcomes.

## ACKNOWLEDGMENTS

This review was supported by the National Natural Science Foundation of China (82341213), CAMS Innovation Fund for Medical Sciences (2021-I2M-1-041), and the Ministry of Science and Technology of China (2021YFC2500304).
